# Supplementary material for: Source identification and distribution reveals the potential of the geochemical Antarctic sea ice proxy IPSO25
Source: Nat Commun. 2016 Aug 30;7:12655. doi: 10.1038/ncomms12655 (PMC5013606; doi:10.1038/ncomms12655)
Supplement: Supplementary Information — Supplementary Figure 1 and Supplementary Table 1 [file ncomms12655-s1.pdf]

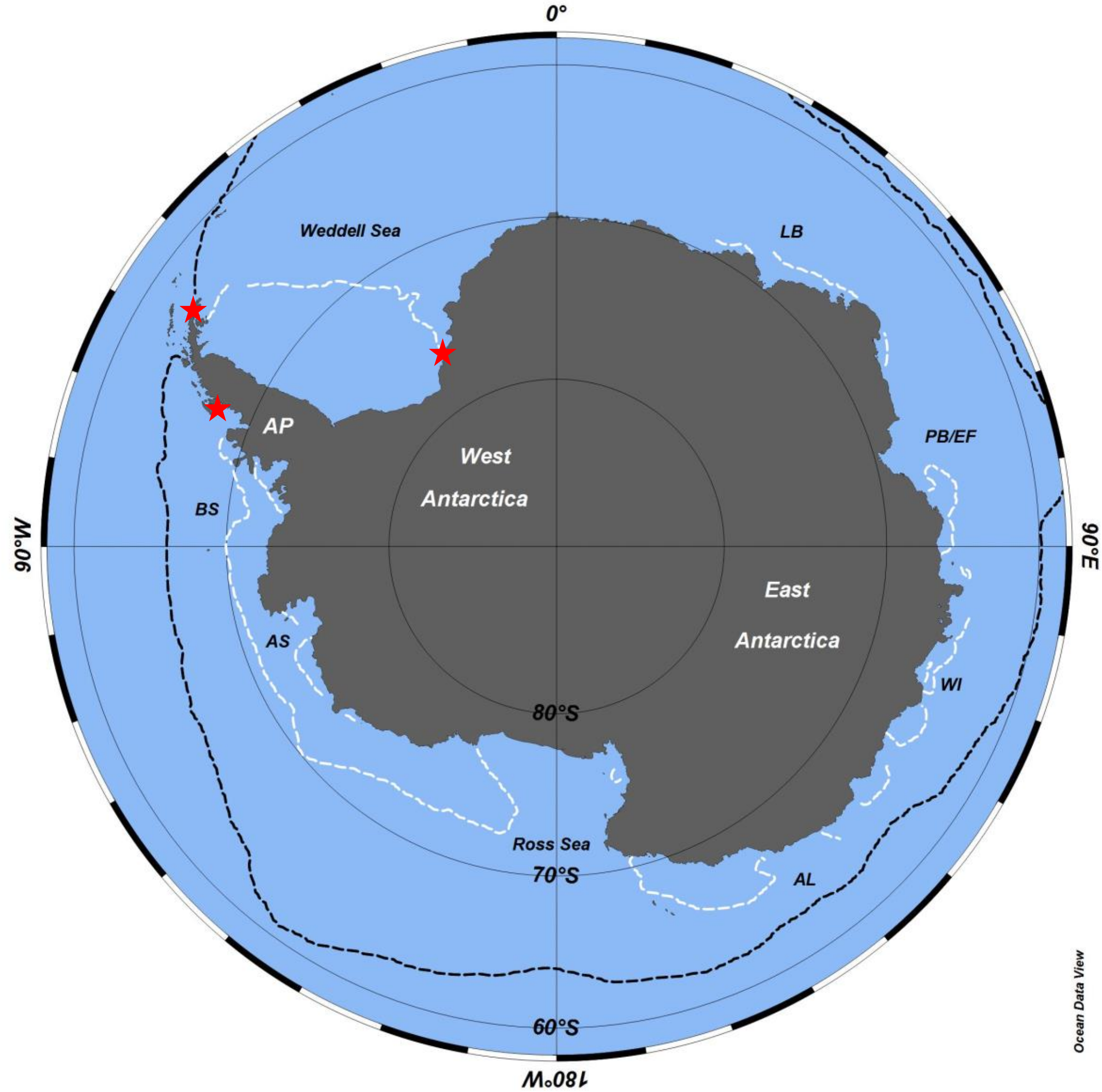

Supplementary Figure 1. **Sample locations for δ^{13}C determinations**. The sample locations for which δ^{13}C data for $IPSO_{25}$ were obtained are indicated with red stars.

Supplementary Table 1. **Core names, locations and $IPSO_{25}$ data**. Concentrations of $IPSO_{25}$ in surface sediments from coastal and near-coastal Antarctic locations.

Station ID	Latitude	Longitude	$IPSO_{25}$ (ng g^{-1})
BC 312	-64.15	-58.53	739.4
BC 313	-64.22	-58.30	1201.4
BC 314	-64.22	-58.48	836.9
BC 315	-64.29	-58.60	348.5
BC 316	-64.38	-58.51	396.2
BC 317	-64.57	-58.49	153.8
BC 319	-64.69	-59.44	99.6
BC 320	-64.83	-60.01	78.4
BC 321	-64.96	-60.24	295.5
BC 351	-70.09	-86.19	0.8
BC 361	-71.99	-76.55	3.3
BC 363	-72.60	-80.83	2.3
BC 364	-72.98	-83.44	3.9
BC 369	-71.58	-82.86	2.0
BC 373	-70.61	-86.25	0.8
BC 379	-69.77	-87.42	0
BC 398	-71.40	-113.39	2.0
BC 403	-71.60	-113.29	1.4
BC 407	-73.21	-115.24	6.9
BC 409	-73.80	-112.82	4.5
BC 412	-73.92	-115.86	16.2
BC 416	-74.14	-112.45	5.2
BC 420	-74.14	-112.86	4.2
BC 421	-73.62	-113.71	2.0
BC 423	-73.45	-115.20	10.4
BC 426	-73.67	-114.98	5.9
BC 429	-73.14	-115.70	11.6
BC 431	-72.30	-118.16	4.2
BC 439	-71.60	-113.30	1.9
BC 443	-71.28	-113.46	0
BC 455	-71.07	-105.08	1.2
BC 476	-74.48	-104.42	53.4
BC 477	-74.36	-104.67	54.5
BC 483	-73.99	-107.38	37.4
BC 485	-72.73	-107.29	6.1
BC 487	-71.18	-109.90	3.6
BC 490	-69.59	-117.98	0
BC 492	-71.15	-119.96	0.4
BC 516	-68.79	-69.88	49.1
BC 518	-68.24	-70.20	34.3
BC 519	-68.24	-70.20	34.1
BC 521	-67.79	-68.08	612.3
BC 523	*-67.86*	*-68.20*	*750.6*
BC 566	-77.27	-33.45	42.2
BC 571	-78.15	-43.65	13.6
BC 574	-78.00	-42.50	2.1
BC 577	-77.65	-42.10	6.9
BC 580	-77.73	-42.17	2.0
BC 584	-77.91	-39.15	3.7
BC 590	-77.61	-38.72	29.6
BC 591	-77.77	-39.39	13.8
BC 605	-77.18	-34.16	28.1
BC 607	-76.79	-30.57	448.8
BC 608	*-76.79*	*-30.57*	*1108.4*
BC 612	-76.42	-29.81	54.4
BC 615	-76.47	-29.69	92.6
BC 621	-76.15	-32.04	50.7
BC 623	-76.47	-31.13	37.8

BC 624	-76.34	-30.28	129.6
BC 627	-76.05	-27.15	25.6
Station ID	Latitude	Longitude	$IPSO_{25}$ (ng g^{-1})
BC 628	-76.02	-26.91	29.4
BC 639	*-63.57*	*-57.29*	*685.5*
BC04-BS05	-61.63	-56.22	12.7
BC06-DP05	-60.69	-63.96	0
BC08-DP03	-60.23	-58.86	0
BF10-BC01	-62.72	-57.88	119.4
BF10-BC02	-62.60	-58.12	55.8
BF10-BC03	-62.19	-57.32	116.0
EAP13-BC16	-66.07	-60.46	24.3
GC 112	-64.68	-70.54	0
GC 513	-68.79	-73.11	14.2
JV10-BC01	-63.13	-54.78	84.6
MC10-BC01	-62.22	-58.79	3.0
MC10-BC02	-62.21	-58.77	1.5
PS 1138-8	-62.27	-57.65	50.9
PS 1273-1	-75.17	-27.34	5.3
PS 1277-1	-77.53	-43.66	11.0
PS 1282-1	-73.40	-20.51	4.0
PS 1284-1	-72.51	-17.46	6.0
PS 1345-8	-62.26	-57.55	11.7
PS 1364-1	-67.85	-20.72	0
PS 1366-2	-70.44	-8.42	10.4
PS 1367-1	-72.34	-16.52	27.4
PS 1370-1	-72.05	-17.44	0.5
PS 1371-1	-72.43	-23.63	0
PS 1372-2	-72.21	-16.72	23.4
PS 1373-2	-72.24	-16.88	12.4
PS 1374-2	-72.22	-16.93	5.8
PS 1375-2	-72.17	-17.13	1.8
PS 1376-2	-71.97	-15.30	58.9
PS 1377-1	-69.27	-10.73	0.5
PS 1378-1	-69.43	-10.50	0
PS 1379-1	-69.73	-10.25	0.2
PS 1380-1	-70.01	-9.99	0.4
PS 1386-1	-68.33	-5.62	0
PS 1388-1	-69.03	-5.89	0
PS 1390-1	-69.62	-6.40	0.3
PS 1394-1	-70.09	-6.68	0.5
PS 1395-1	-70.22	-6.98	2.2
PS 1396-1	-76.95	-50.10	10.0
PS 1397-1	-76.88	-50.03	12.0
PS 1398-2	-76.77	-50.57	5.5
PS 1399-1	-76.82	-51.02	8.4
PS 1402-2	-77.48	-34.73	41.4
PS 1406-1	-71.34	-13.42	109.9
PS 1412-1	-71.04	-13.24	4.9
PS 1421-1	-74.67	-33.96	7.5
PS 1453-1	-66.03	-0.86	0
PS 1539-1	-62.67	-57.26	43.1
PS 1540-1	-61.74	-57.90	6.8
PS 1542-1	-61.29	-58.16	0
PS 1544-1	-62.08	-57.65	7.9
PS 1559-1	-64.78	-67.61	0
PS 1635-2	-71.87	-23.45	0
PS 1798-2	-73.70	-27.36	2.0
PS 1802-2	-73.17	-34.67	1.3
PS 1803-2	-67.51	-5.01	0
PS 1805-5	-66.19	35.31	0.3
PS 1812-5	-66.06	33.28	0
PS 1813-5	-64.96	33.63	2.1

PS 1817-5	-67.99	33.19	4.4
PS 1822-1	-66.92	34.30	0.3
Station ID	Latitude	Longitude	$IPSO_{25}$ (ng g^{-1})
PS 1823-1	-65.93	30.83	0
PS 1824-2	-65.93	30.64	0
PS 1825-5	-66.33	8.89	0
PS 1826-2	-65.03	9.18	0
PS 2006-1	-71.40	-24.31	0
PS 2024-3	-69.97	5.91	6.8
PS 2062-2	-69.78	1.45	1.1
PS 2065-1	-69.72	0.56	2.6
PS 2067-1	-70.44	7.00	7.3
RS15-BC16	-75.66	165.48	358.6
RS15-BC18	-74.91	164.52	20.4
RS15-BC19	-75.00	163.72	8.5
RS15-BC25	-75.82	165.69	60.7
RS15-BC40	-71.62	-165.29	0
RS15-GC54	-70.17	170.54	3.3
RS15-GC57	-71.54	170.06	1174.9
RS15-GC70	-75.96	164.91	320.8
RS15-GC71	-77.09	168.44	309.7
RS15-GC76	-76.92	163.36	1829.1
RS15-GC78	-76.26	163.48	729.4
RS15-GC82	-76.94	166.29	629.5
RS15-GC84	-76.01	163.46	378.8
RS15-LC48	-68.90	178.16	0
WAP13-BC22	-64.70	-63.02	10.8
WAP13-BC45	-65.75	-64.53	1.0
WAP13-BC46	-65.75	-64.47	1.1
WAP13-BC47	-65.61	-64.76	4.1
